# Stroke-associated pneumonia according to mCDC criteria: impact on prognosis and antibiotic therapy

**DOI:** 10.3389/fneur.2024.1358628

**Published:** 2024-02-28

**Authors:** Neus Rabaneda-Lombarte, Júlia Faura, Garbiñe Ezcurra-Díaz, Marta Olivé-Gadea, Marta Álvarez-Larruy, Diana Vidal-de Francisco, Ana Domínguez-Mayoral, Carla Avellaneda, Mari Mar Freijo, Elena Zapata-Arriaza, Gemma Serrano-Heras, Cristian Alcahut-Rodríguez, Isabel Fernández-Pérez, Francisco Moniche, Soledad Pérez-Sánchez, Mònica Millán, Marta Rubiera, Laura Dorado, Olga Maisterra, Joan Montaner, Alejandro Bustamante

**Affiliations:** ^1^Hospital Universitari and Institut de Recerca Germans Trias i Pujol, Universitat Autònoma de Barcelona, Badalona, Spain; ^2^Vall d'Hebrón Institute of Research, Barcelona, Spain; ^3^Hospital Universitari Vall d’Hebrón, Barcelona, Spain; ^4^Complejo Hospitalario de Jaén, Jaén, Spain; ^5^Hospital Universitario Virgen Macarena, Sevilla, Spain; ^6^Hospital del Mar, Barcelona, Spain; ^7^Hospital Universitario Cruces, Barakaldo, Spain; ^8^Hospital Universitario Virgen del Rocío, Sevilla, Spain; ^9^Complejo Hospitalario Universitario de Albacete, Albacete, Spain

**Keywords:** antibiotics, infections, mCDC criteria, outcome, stroke-associated pneumonia

## Abstract

**Objective:**

The modified Centers for Disease Control and Prevention (mCDC) criteria have been proposed for diagnosing and managing stroke-associated pneumonia (SAP). The objective was to investigate the impact of SAP on stroke outcome depending on whether or not it conforms to mCDC criteria. Our secondary objective was to identify the responsible factors for antibiotic initiation in stroke patients.

**Methods:**

We conducted a prospective, multicenter, observational study of ischemic stroke patients with moderate to severe stroke (NIHSS≥4) admitted within 24 h. For 7 days, mCDC criteria were assessed daily, and infections and antibiotics were recorded. Pneumonias were divided into those fulfilling mCDC criteria (mCDC-SAP) or not (other pneumonias, OPn). The effect of each type of pneumonia on 3-month outcome was evaluated in separated logistic regression models. Factors associated with antibiotic initiation were explored using a random forest analysis.

**Results:**

Of the 342 patients studied, infections were diagnosed in 72 (21.6%), including 39 (11.7%) cases of pneumonia. Of them, 25 (7.5%) fulfilled mCDC criteria. Antibiotics were used in 92% of mCDC-SAP and 64.3% of OPn. In logistic regression analysis, mCDC-SAP, but not OPn, was an independent predictor of poor outcome [OR, 4.939 (1.022–23.868)]. The random forest analysis revealed that fever had the highest importance for antibiotic initiation.

**Interpretation:**

The mCDC criteria might be useful for detecting clinically relevant SAP, which is associated with poor outcomes. Isolated signs of infection were more important for antibiotic initiation than compliance with pre-defined criteria. Therefore, adherence to mCDC criteria might result in antibiotic saving without compromising clinical outcome.

## 1 Introduction

Stroke-associated infections (SAI) and especially pneumonia (SAP) represent a significant medical challenge following stroke. Apart from its high prevalence, which ranges between 3.9 to 44% (1, 2), SAP is considered one of the most relevant post-stroke complications in terms of mortality and morbidity (3–7). It has been estimated that 10% of stroke-related deaths can be attributed to SAP (6), which is also associated with poor functional outcomes (1, 7) and longer inpatient stays (3).

Despite the significant impact of SAP on stroke prognosis, there is considerable variability in the diagnosis of SAP (8, 9). The Pneumonia in Stroke Consensus (PISCES) Group proposed the modified Centers for Disease Control and Prevention (mCDC) criteria for SAP diagnosis (10). It is important to note that the mCDC criteria are based on the previously established CDC criteria for diagnosing pneumonia in general rather than specifically in the context of stroke (11). According to the PISCES group, SAP is the term used to describe a spectrum of lower respiratory tract infections occurring within the first 7 days after stroke onset in non-ventilated patients. The mCDC criteria modify the CDC criteria by making not mandatory the presence of chest radiography changes for diagnosing SAP. Therefore, the mCDC criteria classify SAP as definitive (if all CDC criteria are met) or probable (if typical chest radiography changes are not observed) (10). However, the validity of the mCDC criteria and their impact on clinical outcomes have not yet been determined (10), and studies that have used chest-computed tomography to validate these criteria have shown conflicting results (12, 13).

Furthermore, there is variation in SAP management across different healthcare systems in terms of antibiotic treatment (14). For instance, the European Stroke Organization Guidelines state that preventive antibiotics for SAP are not recommended based on current evidence, and infections after a stroke should be treated with appropriate antibiotics (15). However, such approaches are imprecise and may result in initiating antibiotics in front of non-specific signs of infection, such as fever, impaired level of consciousness, or suspected aspiration. This approach can potentially lead to antibiotic overuse and the development of bacterial resistance (16). While consensus recommendations for antibiotic treatment in SAP have recently been proposed (17), they do not provide specific indications for when to initiate antibiotics in cases of suspected pneumonia. Consequently, the potential use of mCDC criteria as a decision-making tool for initiating antibiotics has not yet been evaluated.

The overall aim of the study was to improve the knowledge about pneumonia in the context of stroke patients, highlighting discrepancies in the diagnostic process of SAP and antibiotic stewardship. The primary objective was to assess the impact of SAP on stroke outcome depending on whether or not it conforms to mCDC criteria. The secondary objective was to identify the responsible factors for antibiotic initiation in stroke patients, including early signs of infection and compliance with mCDC criteria.

## 2 Materials and methods

### 2.1 Study design

The PROTEUS (PROteomics and Transcriptomics in lEUkocyte Subpopulations) study was an observational, prospective, multicenter study conducted from July 2018 to May 2021, designed for biomarker research. The present manuscript reports clinical features of the PROTEUS cohort related to SAP diagnosis and treatment. The study was carried out at eight Stroke Units in Spain, with two located at primary stroke centres and six located at comprehensive stroke centres. Inclusion criteria were acute ischemic stroke within the first 24 h after stroke onset, with baseline National Institutes of Health Stroke Scale (NIHSS) score of ≥4, and providing informed consent. Exclusion criteria were: acute infections or antibiotic use within 2 weeks before stroke, chronic infections, inflammatory diseases, or active cancer. Additionally, the study did not include patients with expected hospital stay shorter than 7 days, such as those transferred for acute-phase therapies.

Enrolment in the study was temporarily halted from March 15, 2020, to June 15, 2020, due to the COVID-19 lockdown. To mitigate the impact of pre-stroke asymptomatic COVID-19 infections on the study, an additional inclusion criterion was implemented after June 2020, requiring patients to have a negative PCR test for SARS-CoV-2 upon admission. The originally planned sample size for the study was 500 patients. However, due to slow recruitment following the COVID-19 pandemic and the end of the study funding period, the study was concluded in May 2021.

Following admission, detailed data on infections, antibiotic usage, and neurological status (NIHSS) were prospectively collected, as well as the information needed to evaluate mCDC criteria (Supplementary Table S1), from the clinical records, in a daily manner for 7 days after the occurrence of stroke. Blinding was not applied. Cases recorded as pneumonias by the treating physicians were subsequently categorized as mCDC-SAP (when mCDC criteria were met) or OPn (for those not meeting mCDC criteria) Both probable and definitive pneumonias based on mCDC criteria were considered for the analyses. Functional outcome was assessed 3 months after stroke using the modified Rankin Scale (mRS). A favorable outcome was defined as an mRS score of ≤2, while a score of >2 indicated a poor outcome.

The study received approval from the Hospital Universitari Vall d’Hebrón Clinical Research Ethics Committee [PR (AG)464–2017] as the coordinating centre and from the institutional review boards of each participating centre. Written informed consent was obtained from all patients or their relatives before inclusion in the study.

### 2.2 Statistical analysis

Statistical analysis was performed using Statistical Packages for Social Sciences, version 24, and RStudio, version 1.4.1106, random forest library. Categorical variables were presented as absolute values and percentages, while continuous variables were reported as medians and interquartile ranges. Factors potentially associated with poor outcomes were initially explored through univariate analysis, employing the Chi-squared test for categorical variables and the Mann–Whitney test for continuous variables. Baseline variables that demonstrated statistical significance or showed a trend (*p* < 0.1) were included as covariates in forward-stepwise logistic regression models, with poor outcome as the dependent variable. Two independent logistic regression models were constructed, to evaluate the prognostic impact of mCDC-SAP and OPn separately. The resulting models were compared in terms of accuracy by assessing the area under the receiver operating characteristic (ROC) curve using the likelihood ratio test.

To determine the key variables influencing antibiotic initiation, we initially conducted a univariate analysis, to identify factors associated with antibiotic initiation. This analysis considered baseline information and clinical covariates related to infections, including those collected to assess compliance with the mCDC criteria (10) (Supplementary Table S1). Specifically, the following criteria were evaluated: fever (>38°C without any other identifiable cause); leukopenia (<4,000 WBC/mm^3^) or leukocytosis (>12,000 WBC/mm^3^); altered mental status (with no other identifiable cause, for adults aged ≥70 years); sputum (new onset or change), increased respiratory secretions, or increased requirements for suctioning; cough, dyspnea, or tachypnea (respiratory rate > 25/min); rales, crackles, or bronchial breath sounds (new onset or worsening); worsening gas exchange (O_2_ desaturation, PaO_2_/FiO_2_ ≤ 240, increased oxygen requirements); ≥2 serial chest radiographs with new or progressive and persistent infiltrate, consolidation, or cavitation (a single chest radiograph was deemed acceptable for patients without underlying pulmonary or cardiac disease). For covariates that were evaluated daily, such as NIHSS, fever, and other infection-related variables, the value recorded on the day of antibiotic initiation was used for patients who received antibiotics. For patients who did not receive antibiotics, the average time of antibiotic initiation (72 h) was used as the reference value.

Variables associated with antibiotic initiation or exhibiting a trend (defined as *p* < 0.1) in the previous analysis were included in a random forest plot. Additionally, a new variable indicating compliance with the mCDC criteria on the day of antibiotic initiation was included. Ancillary test-derived variables, such as chest X-ray or blood culture findings, were not included in the analysis due to missing values (as patients who did not initiate antibiotics were generally not tested). Moreover, antibiotics were initiated in most patients, with positive results in those tests. A sensitivity analysis was conducted, excluding cases with infections other than pneumonia. For all random forest analyses, 500 classification and regression trees were performed. The most informative variables were selected based on their higher mean Gini decrease and importance scores (18).

## 3 Results

### 3.1 Baseline characteristics

**Figure 1:**
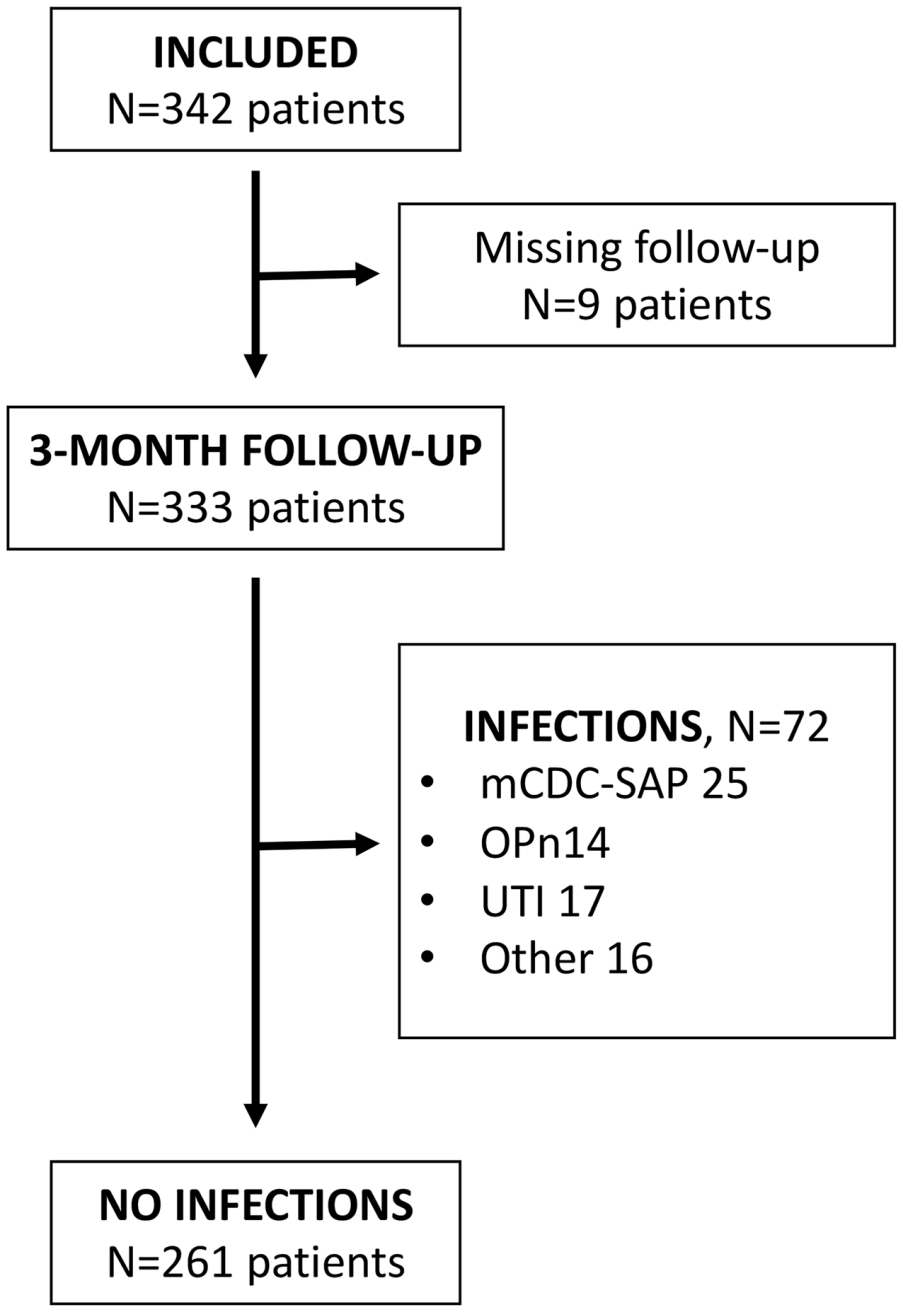
Study flowchart. mCDC-SAP: stroke-associated pneumonia fulfilling mCDC criteria; OPn, other pneumonias not fulfilling mCDC criteria; urinary tract infections.

From July 2018 to May 2021, a total of 342 patients were included in the study. Out of these, functional outcome data at 90 days were available for 333 patients, and these cases were used for further analyses. The demographics of the patients and the incidence of SAI are presented in Table 1. SAI was diagnosed in 72 patients (21.6%), including 39 cases identified as pneumonia (11.7% of the total). Among the pneumonias, 25 cases fulfilled the mCDC criteria, accounting for 7.5% of the cohort and 64.1% of all pneumonias. All of the mCDC-SAP were also recorded as pneumonias by physician’s criteria, as indicated in Table 1. From the 14 cases of OPn, four did not fulfill any of the mCDC criteria and 10 only fulfilled the first mCDC criterion (either fever or leukocytosis) on the day of antibiotic initiation. Median time for mCDC-SAP diagnosis was day 2 (IQR 2–4) and for OPn diagnosis day 2 (1–4). The remaining infections primarily consisted of urinary tract infections (17 cases, 5.1%) and fever of unknown origin (16 cases, 4.8%). Study enrollment and the occurrence of SAIs are illustrated in Figure 1. Antibiotics were administered in 82.1% of pneumonia cases, 92% of mCDC-SAP cases, and 64% of OPn cases. The most commonly prescribed antibiotic was amoxicillin/clavulanic acid, as shown in Table 2.

**Table 1:** Clinical comparison between patients with poor and favorable outcomes.

		Overall *N* = 333	Poor outcome *N* = 182	Favorable outcome *N* = 151	*p*-value
Age		77 (65–84)	81 (69–86)	69 (62–79)	**<0.0001**
Sex (female)		188 (56.5)	91 (50)	97 (64.2)	**0.009**
Hypertension		227 (68.2)	129 (70.9)	98 (64.9)	0.244
Diabetes		99 (29.7)	62 (34.1)	37 (24.5)	**0.057**
Tobacco		78 (23.4)	34 (18.7)	44 (29.1)	**0.025**
Atrial fibrillation		74 (22.2)	53 (29.1)	21 (13.9)	**0.001**
Coronary disease		42 (12.6)	29 (15.9)	13 (8.6)	**0.045**
COPD		35 (10.5)	18 (9.9)	17 (11.3)	0.670
Previous stroke		53 (15.9)	35 (19.2)	18 (11.9)	**0.069**
Previous mRS		0 (0–1)	1 (0–2)	0 (0–0)	**<0.0001**
Baseline NIHSS		10 (5.5–17)	14 (8–20)	6 (4–11)	**<0.0001**
Dysphagia		92 (29.7%)	72 (44.2%)	20 (13.6%)	**<0.0001**
TOAST	Cardioembolic	121 (38.4)	81 (46.8)	40 (28.2)	**0.002**
	Atherothrombotic	72 (22.9)	31 (17.9)	41 (28.9)	
	Lacunar	28 (8.9)	10 (5.8)	18 (12.7)	
	Undetermined	81 (25.7)	46 (26.6)	35 (24.6)	
	Other	13 (4.1)	5 (2.9)	8 (5.6)	
Any infection		72 (21.6)	56 (30.8)	16 (10.6)	**<0.0001**
Any pneumonia		39 (11.7)	33 (18.1)	6 (4.0)	**<0.0001**
mCDC-SAP		25 (7.5)	23 (12.6)	2 (1.3)	**<0.0001**
OPn		14 (4.2)	10 (5.5)	4 (2.6)	0.198
Antibiotics		57 (16.7)	46 (25.3)	10 (6.6)	<0.0001

Clinical variables for the overall cohort and for patients with poor or favourable outcomes. Categorical variables are reported as absolute numbers (percentage), and continuous variables as median (interquartile range). In bold: variables disclosing statistical significance or trend (*p* < 0.1). COPD: chronic obstructive pulmonary disease; mRS: modified Rankin Scale; NIHSS National Institutes of Health Stroke Scale; TOAST: Trial of Org 101072 in Acute Stroke Treatment; mCDC: modified Centers for Disease Control and Prevention, mCDC-SAP: stroke-associated pneumonia (fulfilling mCDC criteria); OPn: other pneumonias (not fulfilling mCDC criteria).

**Table 2:** Infection type and antibiotic use.

		Antibiotic use *N* (%)	Most used antibiotics (%)
All participants Any infection	333 (100) 72 (21.6)	56 (16.8) 56 (77.8)	Amoxicillin/clavulanic acid (67.9) Cefuroxime (8.9) Ciprofloxacin (5.4) Fosfomycin (5.4) Levofloxacin (3.6) Amoxicillin (1.8) Cephalosporin (1.8) Linezolid (1.8) Piperacillin/tazobactam (1.8) Vancomycin (1.8)

Infection type *N* (%)		Antibiotic use in infected patients *N* (%)	Most used antibiotics (%)
mCDC-SAP	25 (7.5)	23 (92)	Amoxicillin/clavulanic acid (78.3) Amoxicillin (4.3) Cephalosporin (4.3) Ciprofloxacin (4.3) Linezolid (4.3) Vancomycin (4.3)
OPn	14 (4.2)	9 (64.3)	Amoxicillin/clavulanic acid (88.9) Levofloxacin (11.1)
Urinary tract infections	17 (5.1)	16 (94.1)	Amoxicillin/clavulanic acid (31.3) Cefuroxime (31.3) Ciprofloxacin (12.5) Fosfomycin (12.5) Levofloxacin (6.3) Piperacillin/tazobactam (6.3)
Other infections*	16 (4.8)	7 (43.8)	Amoxicillin/clavulanic acid (100)
No infections	261 (78.4)	1 (0.4)	Fosfomycin (100)

The table represents the rates of infections and specific infection types, the rate of antibiotic use in each type of infection and the used antibiotics. Values are provided as absolute numbers (percentages). Percentages in the last column (specific antibiotics) are referred to as the total antibiotic use. mCDC: modified Centers for Disease Control and Prevention; mCDC-SAP: stroke-associated pneumonia fulfilling mCDC criteria; OPn: other pneumonias not fulfilling mCDC criteria.*Other infections corresponded in all cases to fever of unknown origin.

### 3.2 Predictors of poor outcome

Patients with poor outcome (182, 54.7%) were found to be older [81 years (69–86) vs. 69 years (62–79)] and predominantly male (50% vs. 35.8%). They also had higher rates of atrial fibrillation (29.1% vs. 13.9%), pre-stroke disability (mRS 1 vs. mRS 0), dysphagia (44.2% vs. 13.6%), and higher baseline NIHSS [14 (8–20) vs. 6 (4–11)], as shown in Table 1. Unfortunately, for dysphagia the rate of missing values was high in the subgroups of mCDC-SAP and OPn (20 and 14%, respectively), and therefore dysphagia was not included as a covariate in logistic regression analyses. The forward-stepwise logistic regression model (Model 1) which included all covariates associated with poor outcome, in addition to mCDC-SAP, identified age [OR, 1.024 (1.000–1.049)], previous mRS [OR, 2.138 (1.581–2.890)], baseline NIHSS [OR, 1.135 (1.088–1.185)], and mCDC-SAP [OR, 4.939 (1.022–23.868)] as independent predictors of poor outcome. Another forward-stepwise logistic regression model (Model 2), including all covariates associated with poor outcome in addition to OPn, revealed age [OR, 1.025 (1.001–1.049)], previous mRS [OR, 2.173 (1.608–2.936)], and baseline NIHSS [OR, 1.145 (1.098–1.195)] as independent predictors of poor outcome, with OPn being not included into the final model (Table 3). Model 1, which included mCDC-SAP, demonstrated improved predictive accuracy compared to Model 2 for the prediction of poor outcome (area under the ROC curve 0.828 (0.784–0.871) vs. 0.822 (0.778–0.867), *p* = 0.021) (Supplementary Figure S1). There were no significant differences in demographics and baseline characteristics between patients with mCDC-SAP and OPn (Supplementary Table S2).

**Table 3:** Forward-stepwise logistic regression models for poor outcome.

	OR (95% CI)	*p*-value
MODEL 1		
Age	1.024 (1.000–1.049)	0.048
Previous mRS	2.138 (1.581–2.890)	<0.0001
Baseline NIHSS	1.135 (1.088–1.185)	<0.0001
mCDC-SAP	4.939 (1.022–23.868)	0.047
MODEL 2		
Age	1.025 (1.001–1.049)	0.043
Previous mRS	2.173 (1.608–2.936)	<0.0001
Baseline NIHSS	1.145 (1.098–1.195)	<0.0001

Odds ratios, 95% confidence intervals and p-values for variables in forward-stepwise logistic regression models for poor outcome. Only significant covariates in the final step of the model are shown. Model 1 includes all covariates associated with poor outcomes in univariate analysis at a *p*-value <0.1 (age, sex, diabetes, tobacco, atrial fibrillation, coronary disease, previous stroke, previous mRS, baseline NIHSS, TOAST) plus mCDC-SAP. Model 2 includes all covariates associated with poor outcomes in univariate analysis at a p-value <0.1 (age, sex, diabetes, tobacco, atrial fibrillation, coronary disease, previous stroke, previous mRS, baseline NIHSS, TOAST) plus OPn. mRS: modified Rankin Scale; NIHSS National Institutes of Health Stroke Scale; mCDC: modified Centers for Disease Control and Prevention, mCDC-SAP: stroke-associated pneumonia fulfilling mCDC criteria; OPn: other pneumonias (not fulfilling mCDC criteria).

### 3.3 Factors associated with antibiotic initiation

**Figure 2:**
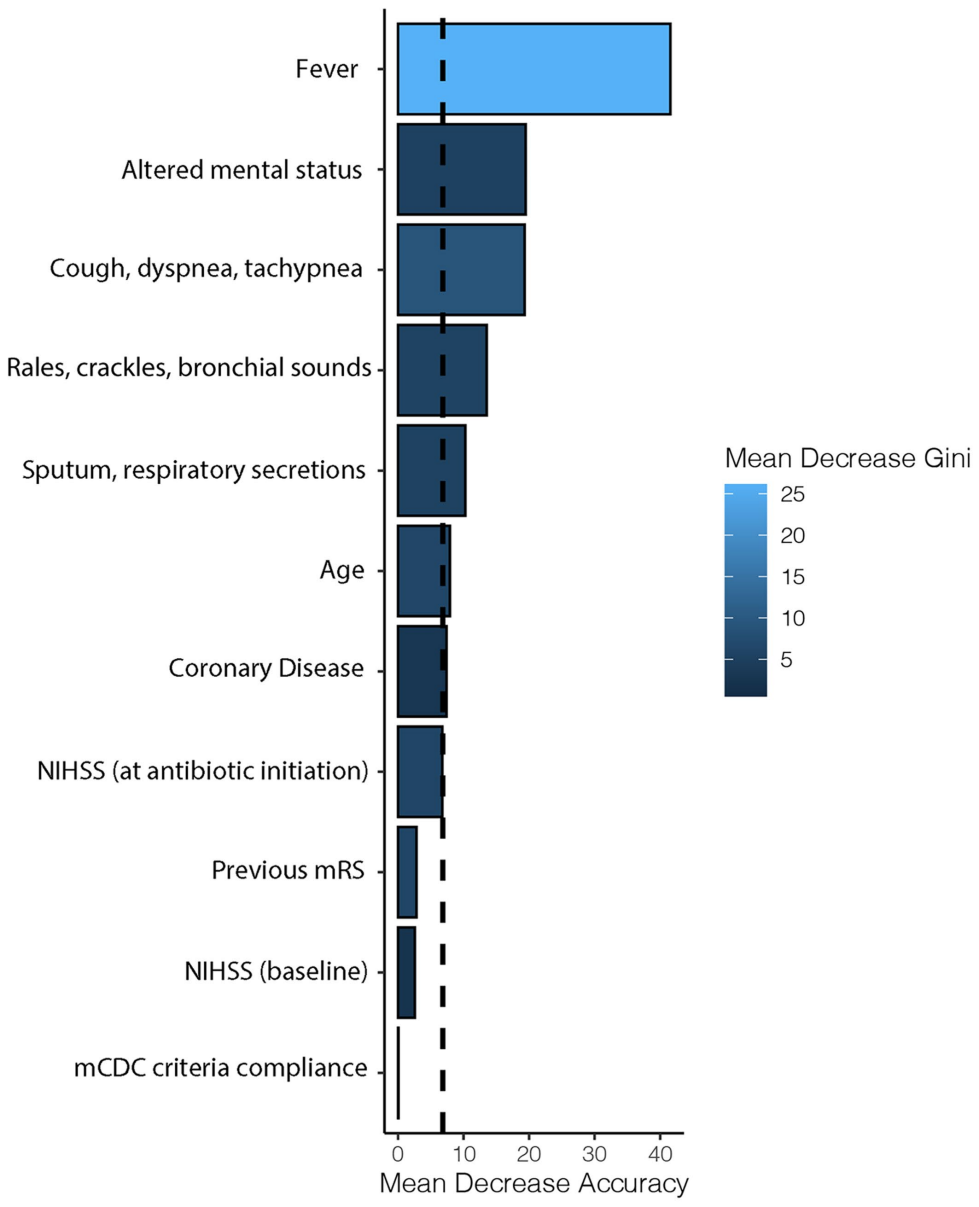
Importance of factors determining antibiotic initiation. Horizontal bars represent the importance of each covariate in terms of mean decrease accuracy (how the accuracy of the model is decreased when the covariate is excluded), while the intensity of the blue color represents the mean decrease Gini (the average gain of purity by splits of the covariate). The dashed line represents the mean decrease accuracy of the model. NIHSS, national institutes of health stroke scale; mRS: modified Rankin Scale; mCDC: modified Centers for Disease Control and Prevention.

**Figure 3:**
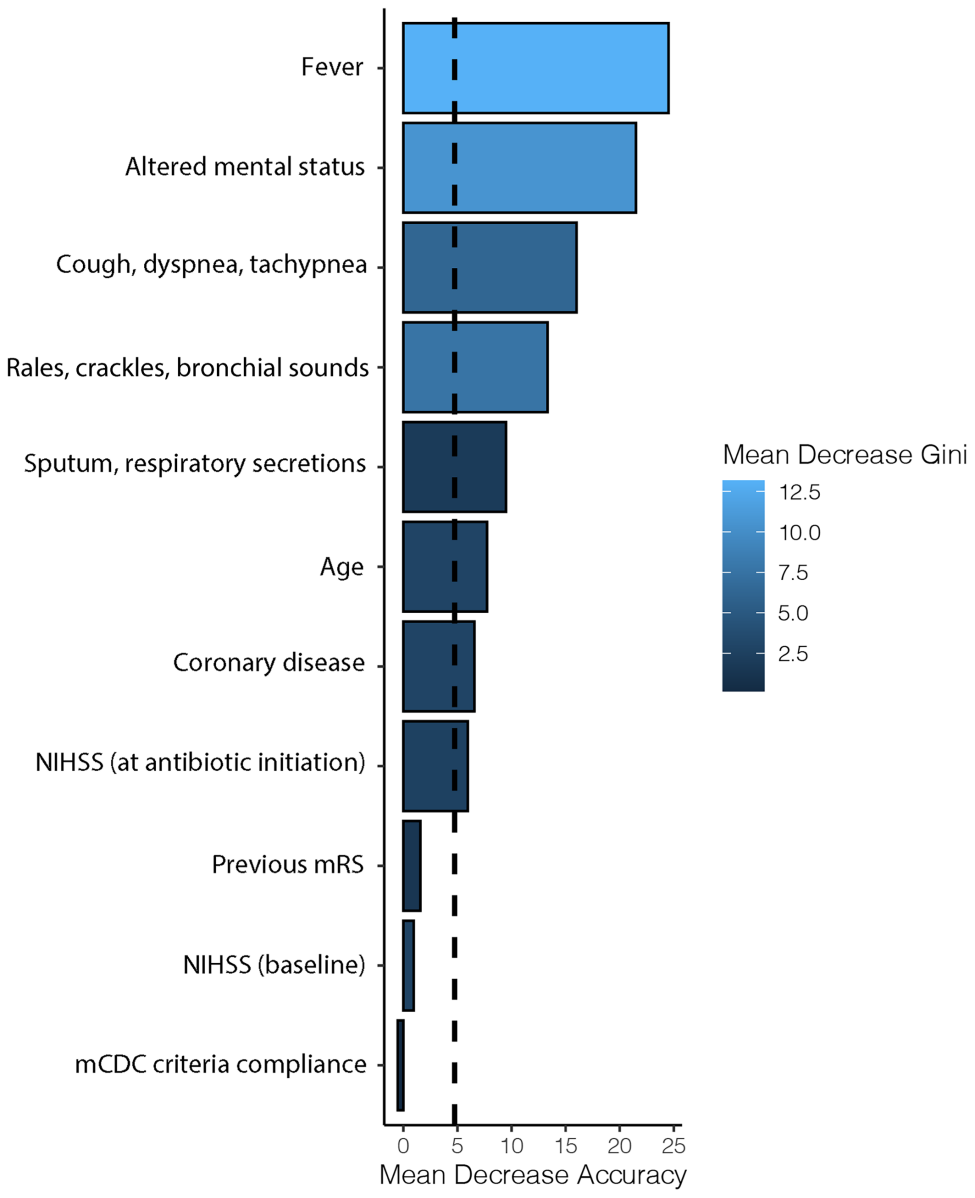
Sensitivity analysis on the importance of factors determining antibiotic initiation after exclusion of infections other than pneumonia. Horizontal bars represent the importance of each covariate in terms of mean decrease accuracy (how the accuracy of the model is decreased when the covariate is excluded), while the intensity of the blue color represents the mean decrease Gini (the average gain of purity by splits of the covariate). The dashed line represents the mean decrease accuracy of the model. NIHSS, National Institutes of Health Stroke Scale; mRS, modified Rankin Scale; mCDC, modified Centers for Disease Control and Prevention.

Regarding factors associated with the initiation of antibiotics (Table 4), the random forest plot (Figure 2) indicated that fever was the most important covariate, followed by other signs of infection (such as altered mental status, cough-dyspnea-tachypnea, auscultatory findings, and sputum-respiratory secretions). Non-specific infectious covariates like NIHSS or age were of lesser importance. Compliance with mCDC criteria was found to have the lowest importance (Figure 2). Similar results were observed when infections other than pneumonias were excluded (Figure 3).

**Table 4:** Factors associated with antibiotic initiation.

	All (*N* = 333)	Antibiotics (*N* = 56)	No antibiotics (*N* = 277)	*p*-value
Age	77 (65–84)	80.5 (69.5–85)	75 (64–83)	**0.027**
Sex (female)	188 (56.6)	28 (50.0)	160 (57.8)	0.285
Hypertension	227 (68.2)	41 (73.2)	186 (67.1)	0.374
Diabetes	99 (29.7)	21 (37.5)	78 (28.2)	0.163
Tobacco	78 (23.4)	11 (19.6)	67 (24.2)	0.464
Atrial fibrillation	74 (22.2)	16 (28.6)	58 (20.9)	0.210
Coronary disease	42 (12.6)	11 (19.6)	31 (11.2)	0.082
COPD	35 (10.5)	9 (16.1)	26 (9.4)	0.302
Previous stroke	53 (15.9)	11 (19.6)	42 (15.2)	0.403
Previous mRS	0 (0–1)	1 (0–2)	0 (0–1)	**0.041**
NIHSS (baseline)	10 (5.5–17)	15.5 (8–22.5)	9 (5–16.5)	**<0.0001**
NIHSS (antibiotic initiation)	6 (2–11)	12 (6.5–19)	5 (2–9)	**<0.0001**
Fever	55 (16.5)	44 (78.6)	11 (4.0)	**<0.0001**
Altered mental status	12 (4.2)	11 (19.6)	3 (1.1)	**<0.0001**
Leukocytosis	15 (4.5)	10 (17.9)	5 (1.8)	**0.008**
Leukopenia	0 (0.0)	0 (0.0)	0 (0.0)	-
Sputum, respiratory secretions	21 (6.3)	18 (32.1)	3 (1.1)	**<0.0001**
Cough, dyspnea, tachypnea	23 (6.9)	20 (35.7)	3 (1.1)	**<0.0001**
Rales, crackles, bronchial sounds	25 (7.5)	21 (37.5)	4 (1.4)	**<0.0001**
Worsening gas exchange	8 (2.4)	8 (14.3)	0 (0.0)	**<0.0001**
Urine Leukocytes	19 (5.7)	18 (32.1)	1 (0.4)	**<0.0001**
Urine nitrites	10 (3.0)	10 (17.9)	0 (0.0)	**<0.0001**
Infiltrate (CXR)	6 (1.8)	6 (10.7)	0 (0.0)	**0.004**
Consolidation (CXR)	4 (1.2)	4 (7.1)	0 (0.0)	**0.027**
Cavitation (CXR)	0 (0.0)	0 (0.0)	0 (0.0)	-
Any positive culture	15 (4.5)	15 (26.8)	0 (0.0)	**<0.0001**

The table represents clinical variables for the overall cohort and for patients receiving antibiotics or not. Categorical variables are reported as absolute numbers (percentage), and continuous variables as median (interquartile range). For covariates that were evaluated daily, values on the day of antibiotic initiation are reported. In patients not receiving antibiotics, information obtained on day 3 (median time for antibiotic initiation) was used. In bold: variables disclosing statistical significance (*p* < 0.05). COPD: chronic obstructive pulmonary disease; mRS: modified Rankin Scale; NIHSS National Institutes of Health Stroke Scale; CXR: chest X-ray.

## 4 Discussion

The main findings of the present study can be summarized as follows: first, SAP had an independent prognostic impact on stroke patients only when diagnosis of SAP was made in accordance with pre-defined criteria (mCDC criteria), and not when diagnosis was exclusively based on clinical judgment. Second, antibiotics in stroke patients were initiated more often due to isolated signs of infection, especially fever, rather than in compliance with pre-defined criteria such as those proposed by the PISCES group.

Consistent with previous studies (19), SAI was a prevalent complication, affecting up to 21.6% of patients, with SAP being the most frequently observed form of SAI, accounting for 11.7% of cases. It is worth noting that the reported prevalence of SAP varies across previous studies, ranging from as low as 1% to as high as 44% (1, 2). This discrepancy may be attributed to the existing variability in SAP diagnosis (9), which emphasizes the importance of well-defined criteria for SAP diagnosis to enhance clinical practice and research. Our study revealed that only 64.1% of diagnosed pneumonias in stroke patients met the criteria for SAP defined by the PISCES group. The fact that almost half of physician’s diagnoses of pneumonia were OPn suggests that physicians are not using mCDC criteria when diagnosing SAP in clinical practice, potentially leading to an overestimation of the actual incidence of SAP in stroke units.

Regarding the outcomes, while most studies identified both SAI and SAP as significant factors associated with poor outcomes (3–7), some reports did not find such associations (20, 21). These discrepancies may arise from variations in the diagnostic approaches utilized. In our study, the clinical outcomes differed between patients who met the mCDC criteria and those who did not. Pneumonias diagnosed according to the mCDC criteria, but not OPn, were identified as independent predictors of poor outcomes. With this comparison of patients who met the pre-defined criteria for SAP with those who did not, we could ascertain that those fulfilling the criteria, likely representing true SAP cases, exhibit this unfavorable outcome. Although speculative, this feature indicates that mCDC criteria may be valuable in identifying clinically relevant SAP cases, where antibiotic treatment may be initiated.

In regard to antibiotic usage, in our study the presence of fever and other isolated signs of infection played a more significant role in initiating antibiotic treatment than adherence to the mCDC criteria. Although the rates of antibiotic use were higher in mCDC-defined SAP (mCDC-SAP) compared to other pneumonias (OPn), the rate of antibiotic use in OPn was not negligible. This suggests that initiating antibiotics based on a criteria-based diagnosis of SAP is not consistently followed and it may be a potential issue of antibiotic overuse. It is not surprising, considering the lack of consensus on the timing of antibiotic initiation for suspected SAP (15, 17), which may lead to variability between healthcare centres and physicians (14). We conducted a random forest analysis to explore the factors associated with antibiotic initiation. Interestingly, the analysis highlighted the higher importance of isolated signs of infection, such as fever, over adherence to mCDC criteria. To ensure the robustness of our findings, we performed a sensitivity analysis excluding cases of other infections, such as urinary tract infections, and confirmed that these results were not confounded by antibiotic treatment for other conditions. The fact that mCDC-SAP emerged as an independent predictor of poor outcomes, while OPn did not, could inform future strategies to optimize antibiotic use in stroke patients. In this context, it seems reasonable to consider delaying antibiotic administration for suspected SAP until the mCDC criteria are fulfilled. OPn may correspond to mild respiratory infections not clinically relevant, maybe viral or even inflammation without infection. These entities may not benefit from antibiotic use and, therefore, mCDC-SAP fulfillment may be a criterion for antibiotic saving without compromising outcomes. However, it is important to note that our study is observational in nature, and we cannot rule out the possibility that the observed improved outcomes in OPn cases might be attributed to earlier antibiotic initiation. Nevertheless, the lack of efficacy of preventive antibiotics in SAP (22) does not support this hypothesis.

In summary, the present study highlights a significant gap in current knowledge regarding the optimal method for diagnosing SAP and the need for additional factors to guide antibiotic use decisions. The variability observed in SAP diagnosis, treatment, and outcomes (8, 9) cannot be solely explained by patient-level clinical characteristics (8), suggesting the involvement of other contributing factors, particularly the diagnostic process. Therefore, there is a clear need for improvement in this area. In this regard, incorporating the mCDC criteria into clinical practice and research studies could play a crucial role in standardizing patient management and enhancing the consistency of research findings.

Strengths of our study include its prospective and multicenter design, the accurate diagnosis of SAP using mCDC criteria, which was assessed in parallel with the information collected by treating physicians, and the utilization of machine learning techniques for statistical analysis. However, there are certain limitations to consider. Firstly, the sample size of the study is relatively small. This is important regarding the logistic regression; in fact, we could not include important covariates such as dysphagia, given relatively high percentages of missing data in patients with mCDC-SAP and OPn. Additionally, mild strokes and intracranial hemorrhages were excluded from the study. Moreover, due to the study’s observational nature, it is impossible to establish causal effects. Furthermore, certain factors that could potentially influence clinical outcomes, such as reperfusion therapies, were not considered.

In conclusion, our findings underscore the importance of employing pre-defined criteria for diagnosing SAP, as meeting these criteria is associated with distinct functional outcomes following stroke. Notably, isolated signs of infection, particularly fever, appear to be the primary driver for initiating antibiotic treatment, outweighing the importance of adhering to the pre-defined criteria. Future studies should investigate the effectiveness of using mCDC criteria as a guide for initiating antibiotics, focusing on optimizing antibiotic usage without compromising clinical outcomes.

## Data availability statement

The raw data supporting the conclusions of this article will be made available by the authors, without undue reservation.

## Ethics statement

The studies involving humans were approved by the Hospital Universitari Vall d’Hebrón Clinical Research Ethics Committee [PR (AG)464-2017]. The studies were conducted in accordance with the local legislation and institutional requirements. The participants provided their written informed consent to participate in this study.

## Author contributions

NR-L: Formal analysis, Writing – original draft, Writing – review & editing. JF: Investigation, Writing – review & editing. GE-D: Investigation, Writing – review & editing. MO-G: Investigation, Writing – review & editing. MÁ-L: Investigation, Writing – review & editing. DF: Investigation, Writing – review & editing. AD-M: Investigation, Writing – review & editing. CA: Investigation, Writing – review & editing. MF: Investigation, Writing – review & editing. EZ-A: Investigation, Writing – review & editing. GS-H: Investigation, Writing – review & editing. CA-R: Investigation, Writing – review & editing. IF-R: Investigation, Writing – review & editing. FM: Investigation, Writing – review & editing. SP-S: Investigation, Writing – review & editing. MM: Conceptualization, Writing – review & editing. MR: Investigation, Writing – review & editing. LD: Investigation, Writing – review & editing. OM: Investigation, Writing – review & editing. JM: Conceptualization, Writing – review & editing. AB: Conceptualization, Supervision, Formal analysis, Writing – review & editing.
