# Supplementary material for: Stroke-associated pneumonia according to mCDC criteria: impact on prognosis and antibiotic therapy
Source: Front Neurol. 2024 Feb 28;15:1358628. doi: 10.3389/fneur.2024.1358628 (PMC10941756; doi:10.3389/fneur.2024.1358628)
Supplement: Supplementary file 1 [file Data_Sheet_1.docx]

Supplementary Material

# Supplementary Data

**Supplemental tables**

- **Table S1: mCDC criteria**
- **Table S2: Comparison of medical history, NIHSS and TOAST classification between patients with mCDC-SAP or other pneumonias.**

**Supplemental Figures**

- **Figure S1: ROC curve analysis**

**SUPPLEMENTAL TABLES**

**Supplemental table S1: Modified Centers for Disease Control and Prevention (mCDC) criteria.** From Smith et al. 2015.

| **At least 1 of the following:** |
| --- |
| 1. Fever (>38°C) with no other recognized cause  2. Leukopenia (<4000 WBC/mm3) or leukocytosis (>12 000 WBC/mm3)  3. For adults ≥70 y old, altered mental status with no other recognized cause |
| **And at least 2 of the following:** |
| 1. New onset of purulent sputum, or change in character of sputum over a 24 h period, or increased respiratory secretions, or increased suctioning requirements  2. New onset or worsening cough, or dyspnea, or tachypnea (respiratory rate>25/min)  3. Rales, crackles, or bronchial breath sounds  4. Worsening gas exchange (eg, O2 desaturation [eg, PaO2/FiO2≤240], increased oxygen requirements*) |
| **And ≥2 serial chest radiographs† with at least 1 of the following:** |
| New or progressive and persistent infiltrate, consolidation, or cavitation  Note: In patients without underlying pulmonary or cardiac disease, 1 definitive chest radiograph is acceptable |

**Probable SAP:** all CDC criteria met, BUT initial CXR and serial/repeat CXR nonconfirmatory (or not undertaken), and no alternative diagnosis or explanation.

**Definite SAP:** ALL CDC criteria met, including diagnostic CXR changes (on at least one).

CXR: chest x-ray; FiO_2_: fraction of inspired oxygen; PaO_2_: partial pressure oxygen; SAP: stroke-associated pneumonia; and WBC: white blood cell.

*Category of increased ventilator demand removed.

†CDC recommendation is for repeat CXR at days 2±7 if initial CXR negative.

**Supplemental table S2:** **Comparison of medical history, NIHSS and TOAST classification between** **patients with mCDC-SAP or other pneumonias.**

|  | | **Overall**  n=39 | **mCDC-SAP**  n=25 | **OPn**  n=14 | **p-value** |
| --- | --- | --- | --- | --- | --- |
| **Age** | | 80.5 (85-69) | 81 (69-85) | 75 (85-67) | 0.714 |
| **Female** | | 21 (53.8) | 15 (60) | 6 (42.9) | 0.303 |
| **Hypertension** | | 30 (76.9) | 20 (80) | 10 (71.4) | 0.542 |
| **Diabetes** | | 16 (41) | 11 (44) | 5 (35.7) | 0.614 |
| **Tobacco** | | 7 (17.9) | 5 (20) | 2 (14.3) | 0.656 |
| **Atrial fibrillation** | | 10 (25.6) | 8 (32) | 2 (14.3) | 0.224 |
| **Coronary disease** | | 8 (20.5) | 6 (24) | 2 (14.3) | 0.471 |
| **COPD** | | 9 (23.1) | 5 (20) | 4 (28.6) | 0.542 |
| **Previous stroke** | | 8 (20.5) | 7 (28) | 1 (7.1) | 0.122 |
| **Previous mRS** | | 1 (0-2) | 1 (0-2) | 0 (0-2) | 0.417 |
| **Baseline NIHSS** | | 17 (11-22) | 19 (13-23) | 14 (7-17) | 0.085 |
| **TOAST** | **Cardioembolic** | 19 (51.4) | 15 (62.5) | 4 (30.8) | 0.119 |
|  | **Atherothrombotic** | 5 (13.5) | 3 (12.5) | 2 (15.4) |  |
|  | **Lacunar** | 1 (2.7) | 0 (0) | 1 (7.7) |  |
|  | **Undetermined** | 10 (27) | 6 (25) | 4 (30.8) |  |
|  | **Other** | 2 (5.4) | 0 (0) | 2 (15.4) |  |

Clinical variables for the overall cohort and for patients with mCDC-SAP or other pneumonias. Categorical variables are reported as absolute number (percentage) and continuous variables as median (interquartile range). In bold: variables disclosing statistical significance or trend (p<0.05). COPD: chronic obstructive pulmonary disease; mRS: modified Rankin Scale; NIHSS National Institutes of Health Stroke Scale; TOAST: Trial of Org 101072 in Acute Stroke Treatment; mCDC: modified Centers for Disease Control and Prevention, mCDC-SAP: stroke-associated pneumonia (fulfilling mCDC criteria); OPn: other pneumonias (not fulfilling mCDC criteria).

**Supplemental figure S1:** **ROC curve analysis.**

**
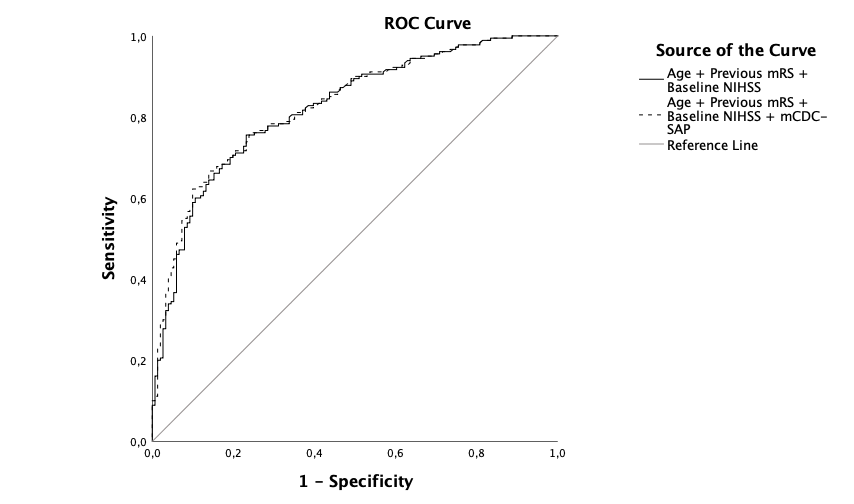
**

The continuous line represents the predictive probabilities for model 2, which included age, previous mRS and baseline NIHSS. The discontinuous line represents the predictive probabilities of model 1, which included the same covariates plus mCDC-SAP. Model 1 improved the predictive accuracy of model 2 for the prediction of poor outcome (area under the ROC curve 0.828 (0.784-0.871) vs. 0.822 (0.778-0.867), p=0.021). NIHSS: National Institutes of Health Stroke Scale; mRS: modified Rankin scale; mCDC-SAP: stroke-associated pneumonia diagnosed according to modified CDC criteria.

# Supplemental References

Smith, C. J. *et al.* (2015) ‘Diagnosis of Stroke-Associated Pneumonia: Recommendations From the Pneumonia in Stroke Consensus Group’, *Stroke; a journal of cerebral circulation*, 46(8), pp. 2335–2340. doi: 10.1161/STROKEAHA.115.009617.
